# Supplementary material for: Immune transcriptomic changes in Australian Gulf War veterans
Source: PLoS One. 2025 Dec 3;20(12):e0338198. doi: 10.1371/journal.pone.0338198 (PMC12674549; doi:10.1371/journal.pone.0338198)
Supplement: Attachment — Submitted filename: Editorial Revision.docx [file pone.0338198.s005.docx]

Letter to the Editor

Regarding submission of manuscript: “Immune transcriptomic changes in Australian Gulf War veterans.”

Thank you for your prompt return of the initial review of the submission of the abovementioned manuscript.

The authors note that concerns regarding the similarities between the written methods of manuscript 10.1172/jci.insight.183810 and the current submission were raised. This manuscript, entitled “Immune Exhaustion in ME/CFS and long COVID” is a manuscript written by the authors of the current submission. Recognising the importance of revising the submission, the authors have substantially reworded the manuscript or inserted additional references within the methods to address this overlap whilst maintaining full disclosure of employed methods.

The authors have further amended the manuscript to enhance readability through the introduction and discussion.

For the editor’s convenience, the authors have submitted a revised version of the document as both a track changes version and a clean version.

We sincerely thank you for your review and time.

Regards,

Dr Natalie Eaton-Fitch.

National Centre for Neuroimmunology and Emerging Diseases,

Griffith University, Australia
