# Supplementary material for: Immune transcriptomic changes in Australian Gulf War veterans
Source: PLoS One. 2025 Dec 3;20(12):e0338198. doi: 10.1371/journal.pone.0338198 (PMC12674549; doi:10.1371/journal.pone.0338198)
Supplement: Attachment — Submitted filename: Response to Reviewers.docx [file pone.0338198.s006.docx]

26^th^ August 2025

Response to Reviewers

Dear Editorial Team, PlosOne

Regarding submission of manuscript: PONE-D-25-24742 “Immune transcriptomic changes in Australian Gulf War veterans.”

Thank you for your prompt return of the review for the abovementioned manuscript. The authors return the revisions of the abovementioned manuscript prior to the deadline of August 29^th^.

A total of 16 comments across three reviewers were identified by the author team. Of the 16 comments, 13 were accepted for modifications incorporated in the manuscript. The remaining 3 comments did not suggest direct changes to the manuscript, and therefore, clarification was provided for the reviewer. All changes made are outlined in the following pages accompanied by the revised manuscript (clean and track changes documents). Additional files submitted in this submission include updated figures for resolution and updated supplementary materials.

We sincerely thank you for your review and time.

Regards,

Dr Natalie Eaton-Fitch.

National Centre for Neuroimmunology and Emerging Diseases,

Griffith University, Australia

Reviewers' comments:

Reviewer's Responses to Questions

**Comments to the Author**

1. Is the manuscript technically sound, and do the data support the conclusions?

The manuscript must describe a technically sound piece of scientific research with data that supports the conclusions. Experiments must have been conducted rigorously, with appropriate controls, replication, and sample sizes. The conclusions must be drawn appropriately based on the data presented.

Reviewer #1: Yes

Reviewer #2: Yes

Reviewer #3: Yes

2. Has the statistical analysis been performed appropriately and rigorously?

Reviewer #1: Yes

Reviewer #2: Yes

Reviewer #3: Yes

3. Have the authors made all data underlying the findings in their manuscript fully available?

The [PLOS Data policy](https://aus01.safelinks.protection.outlook.com/?url=http%3A%2F%2Ftrack.editorialmanager.com%2FCL0%2Fhttp%3A%252F%252Fwww.plosone.org%252Fstatic%252Fpolicies.action%2523sharing%2F1%2F010f0198128e90c9-74522da0-dd21-4825-9863-8a2e2dd11e01-000000%2FYoJAtssdTbUhAs89rfuNKkfHCda2zHWhC5nqg7pYqAY%3D219&data=05%7C02%7Cn.eaton-fitch%40griffith.edu.au%7C3bcdf0300fd4445a424a08ddc44ada4b%7C5a7cc8aba4dc4f9bbf6066714049ad62%7C0%7C0%7C638882548421054875%7CUnknown%7CTWFpbGZsb3d8eyJFbXB0eU1hcGkiOnRydWUsIlYiOiIwLjAuMDAwMCIsIlAiOiJXaW4zMiIsIkFOIjoiTWFpbCIsIldUIjoyfQ%3D%3D%7C0%7C%7C%7C&sdata=wMVOVvdNzbkv1%2BFOi8rqTITv3QpmcoWZqGfA30SUFMk%3D&reserved=0) requires authors to make all data underlying the findings described in their manuscript fully available without restriction, with rare exception (please refer to the Data Availability Statement in the manuscript PDF file). The data should be provided as part of the manuscript or its supporting information, or deposited to a public repository. For example, in addition to summary statistics, the data points behind means, medians and variance measures should be available. If there are restrictions on publicly sharing data—e.g. participant privacy or use of data from a third party—those must be specified.

Reviewer #1: Yes

Reviewer #2: No

Reviewer #3: Yes

4. Is the manuscript presented in an intelligible fashion and written in standard English?

PLOS ONE does not copyedit accepted manuscripts, so the language in submitted articles must be clear, correct, and unambiguous. Any typographical or grammatical errors should be corrected at revision, so please note any specific errors here.

Reviewer #1: Yes

Reviewer #2: Yes

Reviewer #3: Yes

**Reviewer #1:**

**Comment 1:**

The article has been modified as per the suggestions and may be considered for publication check the statistical power and generalizability of the finding lacking protein-level or cellular-level validation.

**Author Response to Reviewer 1 Comment 1:**

*Comment accepted and manuscript modified.*

The authors thank Reviewer 1 for their comment and as per their suggestion have checked the statistical power of the findings. Using the R package “RNASeqPower” it was determined that 7 participants per group were required to meet statistical power for samples with lower effect sizes. Effect sizes were determined using R package “effsize” using normalised expression mean intensity values of healthy controls and veterans with GWI and computed log2 fold change. The effect sizes are included below which demonstrate a moderate to large effect size for the majority of significant genes. The effect size values have now been included within the uploaded supplementary material 1 and a statement in the Discussion (lines 426-428) has been included which reads: “Nevertheless, effect sizes calculated for each differentially expressed gene were found to be moderate to large demonstrating a sufficient sample size to support these findings. With further investigations incorporating larger sample sizes and protein-level validation may also be considered for future research.”

While this current investigation did not complete protein or cellular level validation, the findings demonstrate the necessity of further investigations in this area in which additional funding and resources may be leveraged. A sentiment shared in subsequent reviewer comments.

| **Gene** | **Cohen’s d** |
| --- | --- |
| **Upregulated** | |
| *SIGLEC1* | 0.325 |
| *BPI* | 0.298 |
| *MMP9* | 0.927 |
| *RSAD2* | 0.256 |
| *CEACAM1* | 0.731 |
| *IFIT1* | 0.119 |
| *IFIT3* | 0.317 |
| *CXCL1/2/3* | 0.424 |
| *PTGS2* | 0.322 |
| *CEACAM3* | 1.202 |
| *ITGB3* | 0.934 |
| *EGF* | 0.566 |
| *LTBP1* | 0.941 |
| *GREM2* | 0.531 |
| *OAS3* | 0.486 |
| *IL1B* | 0.372 |
| *ELOVL7* | 0.843 |
| *MX1* | 0.427 |
| *TNFAIP3* | 0.643 |
| *FCAR* | 0.791 |
| *MX2* | 0.68 |
| **Downregulated** | |
| *TRDV3* | -0.884 |
| *IGHG1* | -0.302 |
| *TRGV4* | -0.219 |
| *TRDV1* | -0.817 |
| *TRDV2* | -0.966 |
| *IL7* | -0.945 |
| *IGHV4-59* | -0.446 |
| *EHHADH* | -0.945 |
| *IDO1* | -0.731 |
| *CXCR6* | -0.922 |
| *TRGC1* | -0.654 |
| *SESN2* | -0.739 |

**Reviewer #2:**

**Comment 1:**

Overall this is a useful albeit relatively small contribution that I think is a worthwhile addition to the literature. I have a few small suggestions.

Abstract: Consider re-wording “In this study, gene expression analysis investigated immune exhaustion in GWI, for the first time” since immune exhaustion was not actually a signal supported by the data (I realize that the panel is described as such, but that is made evidence in the Methods section of the abstract). Perhaps something like “In this study, we report for the first time expression-based analysis of a panel of 598 immune function-related genes in GWI”?

**Author Response to Reviewer 2 Comment 1:**

*Comment accepted, manuscript modified.*

The authors thank Reviewer 2 for their comment. While a limitation presented in the manuscript was the sample size of the cohort, the authors agree that this does not discount the contribution to the literature within this area. As an area of unmet need in an aging population, research into the pathomechanisms of Gulf War Illness remains critical to address growing public health concerns. Further, sample size calculations suggest that a sample size of seven per cohort was sufficient for statistical power to observe differential gene expression in those identified.

To address the primary suggestion of this comment, the abstract has been amended to read:

Lines 20-21 of the track changes manuscript: “In this novel study, we report gene expression-based analysis of a panel of 785 immune function related gene markers in GWI.”

Please note that the suggested “598” has been corrected to “785” to accurately demonstrate probes included in the Immune Exhaustion panel used. The discrepancy between the number of probes and the panel and the number of analysed genes is due to the exclusion of data that did not meet background thresholds.

**Comment 2:**

Introduction: The section including the statement that “These toxic agents act as potent immunotoxins” should be qualified. The listed agents have many effects, not just immunological, and many of the “agents” listed are actually categories comprising hundreds or thousands of chemicals/agents (eg insecticides, smoke). Different insecticides work by many different mechanisms, as do particulate matter, PAHs, dioxins, metals and other burn pit smoke constituents, so it doesn’t make mechanistic sense to batch them. For example, the authors could either focus more specifically on insecticides to which (to the best of our limited knowledge) GW veterans were most exposed and cite relevant immune-related literature on those, or simply soften/broaden the statement somewhat.

**Author Response to Reviewer 2 Comment 2:**

*Comment accepted, manuscript modified.*

The authors thank Reviewer 2 for their comment and agree that the original introduction of the manuscript was somewhat generalised to categorise chemicals and agents. The authors have briefly expanded their explanation in the introduction guided by the above comments without diverting from the required information to demonstrate rationale for this research. The introduction now reads:

Lines 57-66 of the track changes manuscript: “Current evidence supports the hypothesis that a combination of toxic chemical and environmental agents, including insecticides, smoke from oil-well fires, pyridostigmine bromide (PB) result in a veteran developing GWI [4]. These toxic environmental and chemical agents are found to be statistically associated with immune system function [5,6]. While intracellular mechanisms remain diverse, agents including organophosphates and carbamate insecticides and PB, used prophylactically by veterans, as well as products from oil well fires, including particulate matter, heavy metals and polycyclic aromatic hydrocarbons are linked with chronic inflammation, oxidative stress and neuronal damage [7–10]. Therefore, demonstrating that no single exposure results in the occurrence of GWI. ”

*Included references:*

[4]	White RF, Steele L, O’Callaghan JP, Sullivan K, Binns JH, Golomb BA, et al. Recent research on Gulf War illness and other health problems in veterans of the 1991 Gulf War: Effects of toxicant exposures during deployment. Cortex 2016;74:449–75. https://doi.org/10.1016/j.cortex.2015.08.022.

[5]	Bou Zerdan M, Moussa S, Atoui A, Assi HI. Mechanisms of Immunotoxicity: Stressors and Evaluators. Int J Mol Sci 2021;22:8242. https://doi.org/10.3390/ijms22158242.

[6]	Semwal R, Semwal RB, Lehmann J, Semwal DK. Recent advances in immunotoxicity and its impact on human health: causative agents, effects and existing treatments. Int Immunopharmacol 2022;108:108859. https://doi.org/10.1016/j.intimp.2022.108859.

[7]	Mitra A, Sarkar M, Chatterjee C. Modulation of Immune Response by Organophosphate Pesticides: Mammals as Potential Model. Proc Zool Soc 2019;72:13–24. https://doi.org/10.1007/s12595-017-0256-5.

[8]	Burzynski HE, Macht VA, Woodruff JL, Crawford JN, Erichsen JM, Piroli GG, et al. Pyridostigmine bromide elicits progressive and chronic impairments in the cholinergic anti-inflammatory pathway in the prefrontal cortex and hippocampus of male rats. Neurobiol Stress 2022;18:100446. https://doi.org/10.1016/j.ynstr.2022.100446.

[9]	Joyce MR, Holton KF. Neurotoxicity in Gulf War Illness and the potential role of glutamate. Neurotoxicology 2020;80:60–70. https://doi.org/10.1016/j.neuro.2020.06.008.

[10]	Brooks AW, Sandri BJ, Nixon JP, Nurkiewicz TR, Barach P, Trembley JH, et al. Neuroinflammation and Brain Health Risks in Veterans Exposed to Burn Pit Toxins. Int J Mol Sci 2024;25:9759. https://doi.org/10.3390/ijms25189759.

**Comment 3:**

[Introduction:] The abstract mentions cellular stress and metabolic impacts, and these are discussed in the Results, so I would suggest a brief mention of these in the introduction as well (what we know about how these are altered in GWI, and by deployment-related exposures).

**Author Response to Reviewer 2 Comment 3:**

*Comment accepted, manuscript modified.*

The authors thank Reviewer 2 for their comment, in the drafting of the original manuscript, the introduction focused on immunological disturbances in GWI to provide rationale to conduct this investigation. However, aligning with the suggestions made, the authors have amended the introduction to incorporate background on cellular stress and metabolic impacts in relation to immune cell disturbances in veterans living with GWI. The introduction now reads:

Lines 67-80 of the track changes manuscript: “One mechanism may include the inhibition of acetylcholinesterase by insecticides and PB resulting in an accumulation of acetylcholine which consequentially influences cellular metabolism and immunological functions, including reactive oxygen species (ROS), chronic inflammation, impaired cytotoxic pathways, cytokine production and immune cell activation [7–10]. As an example, an investigation into the effect of the pesticide permethrin with PB on a GWI mice model reported increased activation of both peripheral and brain adaptive immune responses [11]. Further, the accumulation of acetylcholine is further linked with oxidative stress. In an experimental GWI mice model, impaired cellular metabolism promoting ROS have been reported in association with immune dysregulation [16]. This is further evidenced in diverse immunological studies reporting lymphocyte disturbances, altered lymphocyte subsets, interleukin (IL) and cytokine production, and production of antibodies are modified in veterans with GWI [12–15].”

*Included references:*

[7]	Mitra A, Sarkar M, Chatterjee C. Modulation of Immune Response by Organophosphate Pesticides: Mammals as Potential Model. Proc Zool Soc 2019;72:13–24. https://doi.org/10.1007/s12595-017-0256-5.

[8]	Burzynski HE, Macht VA, Woodruff JL, Crawford JN, Erichsen JM, Piroli GG, et al. Pyridostigmine bromide elicits progressive and chronic impairments in the cholinergic anti-inflammatory pathway in the prefrontal cortex and hippocampus of male rats. Neurobiol Stress 2022;18:100446. https://doi.org/10.1016/j.ynstr.2022.100446.

[9]	Joyce MR, Holton KF. Neurotoxicity in Gulf War Illness and the potential role of glutamate. Neurotoxicology 2020;80:60–70. https://doi.org/10.1016/j.neuro.2020.06.008.

[10]	Brooks AW, Sandri BJ, Nixon JP, Nurkiewicz TR, Barach P, Trembley JH, et al. Neuroinflammation and Brain Health Risks in Veterans Exposed to Burn Pit Toxins. Int J Mol Sci 2024;25:9759. https://doi.org/10.3390/ijms25189759.

[11]	Joshi U, Pearson A, Evans JE, Langlois H, Saltiel N, Ojo J, et al. A permethrin metabolite is associated with adaptive immune responses in Gulf War Illness. Brain, Behavior, and Immunity 2019;81:545–59. https://doi.org/10.1016/j.bbi.2019.07.015.

[12]	Shetty GA, Hattiangady B, Upadhya D, Bates A, Attaluri S, Shuai B, et al. Chronic Oxidative Stress, Mitochondrial Dysfunction, Nrf2 Activation and Inflammation in the Hippocampus Accompany Heightened Systemic Inflammation and Oxidative Stress in an Animal Model of Gulf War Illness. Front Mol Neurosci 2017;10. https://doi.org/10.3389/fnmol.2017.00182.

[13]	Whistler T, Fletcher MA, Lonergan W, Zeng X-R, Lin J-M, LaPerriere A, et al. Impaired immune function in Gulf War Illness. BMC Medical Genomics 2009;2:12. https://doi.org/10.1186/1755-8794-2-12.

[14]	Vojdani A, Thrasher JD. Cellular and humoral immune abnormalities in Gulf War veterans. Environ Health Perspect 2004;112:840–6. https://doi.org/10.1289/ehp.6881.

[15]	Broderick G, Kreitz A, Fuite J, Fletcher MA, Vernon SD, Klimas N. A pilot study of immune network remodeling under challenge in Gulf War Illness. Brain Behav Immun 2011;25:302–13. https://doi.org/10.1016/j.bbi.2010.10.011.

[16]	Sultana E, Shastry N, Kasarla R, Hardy J, Collado F, Aenlle K, et al. Disentangling the effects of PTSD from Gulf War Illness in male veterans via a systems-wide analysis of immune cell, cytokine, and symptom measures. Military Medical Research 2024;11:2. https://doi.org/10.1186/s40779-023-00505-4.

**Comment 4:**

Methods: Why were two methods used for RNA extraction? Could this have introduced any bias into the results?

**Author Response to Reviewer 2 Comment 4:**

*Clarification provided, manuscript not modified.*

The authors thank the reviewer for their comment and wish to provide clarification. A small number of samples (n=2) were extracted using the trizol method from a previous research project. Nanodrop readings indicated not only a high yield, but also a high purity. Therefore, any potential risk arising from an alternative method (being reagent contaminants) has been removed. For the reviewer’s interest, the 260/280 and 260/230 readings for the two samples are below demonstrating high purity meeting NanoString requirements:

| **Sample Name** | **260/280** | **260/230** |
| --- | --- | --- |
| HC10 | 1.98 | 1.99 |
| HC11 | 1.90 | 1.23 |

**Comment 5:**

[Methods:] Nanodrop readings indicate concentration and purity (and purity measurement ranges should be reported—ie 260/280 and 230/260 ratios), but not quality in the sense of RNA integrity.

**Author Response to Reviewer 2 Comment 5:**

*Comment accepted, manuscript modified.*

The authors thank the reviewer for their comment and have included mention of the RNA purity within the methods of the manuscript which now reads:

Lines 123-127 of the track changes manuscript: “RNA purity values were recorded with the mean and standard deviation being 1.99 ± 0.10 for 260/280 and 1.33 ± 0.43 for 260/230. While contaminants appeared present according to 260/230 values, all samples passed RNA binding density. The quality of the samples was confirmed using LabChip RNA Standard Sense Assay. All RNA samples returned a quality score above 8.9.”

The 260/280 ratio demonstrate that the RNA was free from protein contamination; however, the 260/230 ratios indicate potential reagent residuals might be present. As the NanoString technique does not require amplification the technique is less sensitive to potential reagent contaminants. All binding density and control performance values passed (as included within the original Supplementary Material) manufacturer QC thresholds for normalisation control performance and thus were included in the analysis.

| **Sample Name** | **260/280** | **260/230** |
| --- | --- | --- |
| GWI01 | 2.15 | 0.87 |
| GWI02 | 1.92 | 1.70 |
| GWI04 | 1.91 | 1.77 |
| GWI05 | 1.96 | 0.73 |
| GWI06 | 1.98 | 1.41 |
| GWI07 | 2.03 | 1.32 |
| GWI08 | 1.98 | 1.58 |
| GWI09 | 1.97 | 2.06 |
| GWI10 | 2.03 | 1.44 |
| GWI11 | 1.96 | 1.63 |
| GWI12 | 2.07 | 1.53 |
| GWI13 | 1.93 | 1.26 |
| GWI14 | 2.26 | 0.90 |
| GWI15 | 2.03 | 2.11 |
| GWI16 | 1.75 | 1.04 |
| GWI17 | 1.99 | 1.87 |
| GWI18 | 1.94 | 1.79 |
| GWI19 | 2.07 | 0.75 |
| GWI20 | 1.96 | 1.35 |
| HC01 | 1.99 | 0.76 |
| HC02 | 1.96 | 0.93 |
| HC03 | 1.97 | 0.59 |
| HC04 | 1.99 | 2.10 |
| HC05 | 1.91 | 1.21 |
| HC06 | 2.02 | 0.88 |
| HC07 | 2.04 | 1.04 |
| HC08 | 2.08 | 1.3 |
| HC09 | 2.31 | 0.92 |
| HC10 | 1.98 | 1.99 |
| HC11 | 1.90 | 1.23 |
| HC12 | 1.88 | 1.53 |
| HC13 | 1.89 | 1.00 |
| HC14 | 2.12 | 1.15 |
| HC15 | 2.01 | 1.38 |

**Comment 6:**

[Methods:] Do the authors have any concerns about age skewing the results, given the lower average ages of the HC individuals (and apparently at least one much younger participant, at 18)?

**Author Response to Reviewer 2 Comment 6:**

*Comment accepted, manuscript modified.*

The authors thank the reviewer for their comment, the authors have conducted an analysis to determine whether age was a predictor of gene expression changes. A general multivariate linear model was used with age as a covariate. The data below demonstrates that the effect of age was non-significant (excluding *IGHV4/59*), thus the fold change is reliable across participant ages.

Included in the limitations highlighted within the discussion reads:

Lines 423-425 of the track changes manuscript: “Given the significant differences in age between the cohorts, a multivariate analysis was performed to determine any potential effect on gene expression. We report that the effect of age on gene expression was non-significant, excluding one gene being *IGHV4/59*.”

| Gene | F | p-value |
| --- | --- | --- |
| TRDV3 | 0.581 | 0.451 |
| TRDV1 | 0.406 | 0.529 |
| TRDV2 | 0.007 | 0.933 |
| TRGC1 | 0.259 | 0.614 |
| TRGV4 | 0.251 | 0.620 |
| IL7 | 0.281 | 0.600 |
| IGHV4/59 | 7.515 | 0.010 |
| IGHG1 | 1.488 | 0.231 |
| EHHADH | 1.295 | 0.264 |
| IDO1 | 1.212 | 0.279 |
| CXCR6 | 0.168 | 0.684 |
| SESN2 | 0.003 | 0.960 |
| SIGLEC1 | 0.061 | 0.806 |
| BPI | 0.080 | 0.825 |
| MMP9 | 2.657 | 0.113 |
| RSAD2 | 0.0 | 0.997 |
| CEACAM1 | 1.917 | 0.176 |
| CEACAM3 | 0.911 | 0.347 |
| IFIT1 | 0.208 | 0.652 |
| IFIT3 | 0.791 | 0.380 |
| CXCL1/2/3 | 0.190 | 0.666 |
| PTGS2 | 0.917 | 0.345 |
| ITGB3 | 0.457 | 0.504 |
| EGF | 0.742 | 0.395 |
| LTBP1 | 2.048 | 0.162 |
| GREM2 | 2.024 | 0.165 |
| OAS3 | 0.182 | 0.673 |
| IL1B | 0.367 | 0.549 |
| ELOVL7 | 0.861 | 0.360 |
| MX1 | 0.126 | 0.725 |
| MX2 | 0.561 | 0.459 |
| TNFAIP3 | 2.391 | 0.132 |
| FCAR | 0.499 | 0.485 |

**Comment 7:**

Results: The authors report SEM instead of SD for population demographic characteristics, I assume because they are comparing these categories statistically. However, since reporting only the SEM and not SD or range makes it difficult to evaluate the population distributions, I recommend also reporting the ranges and/or SDs.

**Author Response to Reviewer 2 Comment 7:**

*Comment accepted, manuscript modified.*

The authors thank reviewer 2 for their comment, the mean, SEM and SD are now provided for all continuous demographic variables. This is represented in the results of the modified manuscript.

**Table 1: Participant demographics.**

|  | **HC** | | | **GWI** | | | **P-value** |
| --- | --- | --- | --- | --- | --- | --- | --- |
|  | N(%) | | | N(%) | | |  |
| Education n (%) |  |  |  |  |  |  | 0.110 |
| Primary School | 0 (0.0) | | | 0 (0.0) | | |  |
| High School | 3 (20.0) | | | 7 (35.0) | | |  |
| Undergraduate | 2 (13.3) | | | 8 (40.0) | | |  |
| Postgraduate | 7 (46.7) | | | 3 (15.0) | | |  |
| Other | 3 (20.0) | | | 2 (10.0) | | |  |
| Employment n (%) |  |  |  |  |  |  | **0.027** |
| Full Time | 11 (73.3) | | | 9 (45.0) | | |  |
| Part Time | 2 (13.3) | | | 0 (0.0) | | |  |
| Casual | 1 (6.7) | | | 1 (5.0) | | |  |
| Unemployed (other) | 1 (6.7) | | | 9 (45.0) | | |  |
| Unemployed illness | 0 (0.0) | | | 1 (5.0) | | |  |
| Sex n (%) |  |  |  |  |  |  |  |
| Male | 15 (100%) | | | 20 (100.0%) | | |  |

Categorical variables compared using chi-square test. Data presented as n (%). *Abbreviations: HC, healthy control; GWI, Gulf War Illness.*

**Table 2: Participant demographics (cont.), full blood analysis and quality of life.**

|  | **HC** | | | **GWI** | | | **P-value** |
| --- | --- | --- | --- | --- | --- | --- | --- |
|  | Mean | SEM | SD | Mean | SEM | SD |  |
| Age | 47.47 | 2.91 | 11.27 | 54.4 | 0.74 | 3.29 | **0.013** |
| BMI | 25.96 | 1.09 | 4.23 | 31.44 | 1.62 | 7.23 | **0.013** |
| Full blood count analysis |  |  |  |  |  |  |  |
| WCC (x10^9^/L) | 6.11 | 0.31 | 1.19 | 6.78 | 0.33 | 1.47 | 0.158 |
| Lymphocyte (x10^9^/L) ^a^ | 1.88 | 0.19 | 0.77 | 2.03 | 0.14 | 0.63 | 0.214 |
| Neutrophils (x10^9^/L) | 3.49 | 0.20 | 0.79 | 3.98 | 0.26 | 1.15 | 0.163 |
| Monocytes (x10^9^/L) | 0.51 | 0.05 | 0.18 | 0.56 | 0.03 | 0.15 | 0.287 |
| Eosinophils (x10^9^/L) ^a^ | 0.19 | 0.04 | 0.14 | 0.16 | 0.02 | 0.09 | 0.856 |
| Basophils (x10^9^/L) | 0.05 | 0.01 | 0.02 | 0.05 | 0.004 | 0.02 | 0.924 |
| Platelets (x10^9^/L) | 260.13 | 10.97 | 42.49 | 263.55 | 14.91 | 66.66 | 0.863 |
| RCC (x10^12^/L) | 5.08 | 0.08 | 0.32 | 5.07 | 0.09 | 0.39 | 0.932 |
| Haematocrit | 0.44 | 0.01 | 0.02 | 0.45 | 0.01 | 0.03 | 0.082 |
| Haemoglobin (g/L) | 149.07 | 2.24 | 8.68 | 151.10 | 2.30 | 10.31 | 0.542 |
|  |  |  |  |  |  |  |  |
| SF-36 |  |  |  |  |  |  |  |
| General Health | 71.38 | 4.60 | 17.81 | 37.25 | 4.12 | 18.44 | **<0.001** |
| Physical Functioning^a^ | 96.0 | 2.02 | 7.83 | 57.50 | 5.99 | 26.83 | **<0.001** |
| Role Physical | 82.92 | 9.46 | 36.63 | 45.31 | 6.44 | 28.81 | **0.002** |
| Role Emotional | 95.56 | 2.68 | 10.38 | 49.99 | 7.13 | 31.88 | **<0.001** |
| Pain^a^ | 90.67 | 3.40 | 13.17 | 38.0 | 4.54 | 20.32 | **<0.001** |
| Mental Health | 83.67 | 3.57 | 13.82 | 47.50 | 5.37 | 24.03 | **<0.001** |
| Vitality | 75.83 | 3.39 | 13.12 | 40.63 | 3.06 | 13.68 | **<0.001** |
| Social Functioning | 97.50 | 1.81 | 7.01 | 43.75 | 6.87 | 30.75 | **<0.001** |
|  |  |  |  |  |  |  |  |
| WHO DAS |  |  |  |  |  |  |  |
| Understanding & communication | 8.06 | 3.56 | 13.78 | 39.79 | 5.17 | 23.12 | **<0.001** |
| Mobility | 2.33 | 1.61 | 6.23 | 33.0 | 5.39 | 24.08 | **<0.001** |
| Self-care^a^ | 0.83 | 0.83 | 3.23 | 15.0 | 3.37 | 15.09 | **<0.001** |
| Relationships | 5.42 | 2.58 | 9.98 | 45.0 | 5.82 | 26.02 | **<0.001** |
| Life activities | 2.08 | 1.45 | 5.62 | 36.56 | 5.29 | 23.67 | **<0.001** |
| Participation in society | 5.21 | 2.77 | 10.74 | 44.37 | 6.26 | 28.01 | **<0.001** |

Continuous variables compared using Mann Whitney U test or T test. *^a^* Denotes continuous variables compared using Mann Whitney U test. The WHO DAS domain for participation in work/school was omitted given the high number of participants reporting unemployment. Data presented as mean, SEM and SD. *Abbreviations: HC, healthy control; GWI, Gulf War Illness; BMI, body mass index; SD, standard deviation; SEM, standard error of mean; WCC, white cell count; RCC, red cell count; SF-36, 36 item short-form health survey; WHO, World Health Organization; DAS, disability assessment schedule.*

**Comment 8:**

[Results:] Are the p values for each gene corrected in any way for multiple comparisons? “This panel contains 785 genes to elucidate mechanisms behind T cell, B cell and NK cell exhaustion in disease.” Is struck out in Track Changes in the manuscript, but I think should be retained—this is useful information that readers may wish to easily access, without going to Suppl File 1. But—now that I have opened that file—it has fewer than 600 genes listed. Why not 785, if this is raw data?

**Author Response to Reviewer 2 Comment 8:**

*Comment accepted, manuscript modified.*

The authors thank the reviewer for their comment. The authors wish to provide clarification regarding the correction of p values. Unadjusted and adjusted p values provided by Rosalind Bio for all investigated genes were included within Supplementary Material 2. For convenience, the false discovery rate (FDR) correction can also be found for the top differentially expressed genes under an isolated excel sheet titled “Statistically Sig genes”. The FDR p-values for the top statistically significant genes remains significant.

Regarding the statement struck out in track changes, the original sentence was removed to avoid repetition with previously published works by the authors, instead referencing the methods undertaken in previous research. To provide clarification on the number of genes, the NanoString Immune Exhaustion panel contains 785 probes; however, as only 601 genes were returned during the analysis, this indicates that 184 genes did not reach background thresholding or normalisation or were of low expression and automatically excluded by the analysis software (Rosalind Bio). A brief sentence has been included in the results of the manuscript to reflect this.

Lines 186-187 of the track changes manuscript: “Of the 785 genes that may be identified by the NanoString Immune Exhaustion Panel, 601 genes passed background thresholding or normalisation.”

**Comment 9:**

[Results:] Figures 2 and 3 were too low resolution for me to read (Figure 1 too, but I think the take-home was obvious). I could more or less evaluate Figure 2 (the color code along the top was unclear), but Figure 3 was illegible.

**Author Response to Reviewer 2 Comment 9:**

*Comment accepted, manuscript modified*.

The authors thank the reviewer for their comment. New figures have been exported for high resolution (300-600dpi). These figures have been uploaded with the new modified manuscript.

**Comment 10:**

Discussion: Similar to my earlier comment about the Abstract, consider rewording “This novel study investigates altered gene expression related to immune exhaustion” to “This novel study investigates altered gene expression related to immune function”

**Author Response to Reviewer 2 Comment 10:**

*Comment accepted, manuscript modified.*

The authors thank the reviewer for their comment and have amended the first line of the discussion according to the above suggestion:

Line 303 of the track changes manuscript: “This novel study investigates altered gene expression related to immune function in Australian veterans with GWI compared with HC.”

**Comment 11:**

The paragraph on mitochondrial/metabolic alterations could be expanded a bit, and discussed in relation to the immune function focus of this manuscript. The possible change in fatty acid metabolism makes me think of these publications: <https://pmc.ncbi.nlm.nih.gov/articles/PMC6660083/> <https://pubmed.ncbi.nlm.nih.gov/37438460/> . Altered ATAD3A levels suggest mitochondrial involvement and engagement of the cGAS STING pathway, thereby relating as well to possible inflammation (eg, <https://pubmed.ncbi.nlm.nih.gov/34333111/> ). There is also a literature on mitochondrial DNA damage and copy number alteration that may be relevant, since this outcome was mentioned in the context of SESN2 downregulation.

**Author Response to Reviewer 2 Comment 11:**

*Comment accepted, manuscript modified*.

The authors thank the reviewer for their comment and have included additional information in the discussion according to the above suggestion. While differentially expressed genes were associated with type I interferon signalling, other genes related to cGAS-STING (*TMEM173*, and *MB21D1* and various kinases), as introduced by the Reviewer, were not found in the present study and therefore not discussed in detail in the modified manuscript. In order to discuss the potential link between inflammatory mediators and metabolic disturbances, the following information has been included in the modified manuscript. Furthermore, the authors have incorporated all three suggested references in the modified manuscript, but also outline variation of data and methodology across research models, being samples obtained directly from veterans with GWI or established GWI mouse models. Nevertheless, the data is indicative of potential metabolic dysfunction and thus provides avenues for future research.

Lines 373-389 of the modified manuscript: “Previous investigations have reported that impaired mitochondrial function is associated with symptom severity in veterans with GWI [41]. A review of research on inflammation and ROS suggests that inflammatory mediators, such as those reported in GWI cohorts, may potentially exacerbate metabolic dysfunction and fatty acid oxidation [42]. Suggesting that inflammation and mitochondrial dynamics are interconnected in disease. Additionally, Bryant *et al.* reported that altered cellular metabolism in a GWI mouse model may promote inflammatory processes in veterans; however, further protein- and functional-level research is required to determine whether this is supported by the present study. The downstream effects of genes including *SESN2* and others listed above on cellular metabolism may indeed exacerbate inflammation aligning with Bryant *et al* [25]*.* Other research has reported on genetic variants linked with mitochondrial disturbances, such as *BChE* [23]. While transcriptomics research in GWI mice have also reported differential expression of genes important for mitochondrial respiration, oxidative phosphorylation and electron transport chain [43], the top differentially expressed genes are not replicated by the present investigation. Differences in the literature may be linked to the use of a GWI mouse model [25,43] or samples provide by veterans with GWI [41,44], therefore, posing a barrier when interpreting data between models. Overall, this present research is supported by the literature reporting a decline in metabolic function in veterans with GWI and therefore provides avenues for future research [44–46].”

*Included references:*

[23]	Golomb BA, Kelley RI, Han JH, Miller B, Bui L. Gulf war illness: a tale of two genomes. BMC Research Notes 2024;17:230. https://doi.org/10.1186/s13104-024-06871-z.

[25]	Bryant JD, Kodali M, Shuai B, Menissy SS, Graves PJ, Phan TT, et al. Neuroimmune mechanisms of cognitive impairment in a mouse model of Gulf War illness. Brain Behav Immun 2021;97:204–18. https://doi.org/10.1016/j.bbi.2021.07.015.

[41]	Golomb BA, Sanchez Baez R, Schilling JM, Dhanani M, Fannon MJ, Berg BK, et al. Mitochondrial impairment but not peripheral inflammation predicts greater Gulf War illness severity. Sci Rep 2023;13:10739. https://doi.org/10.1038/s41598-023-35896-w.

[42]	Nesci S, Spagnoletta A, Oppedisano F. Inflammation, Mitochondria and Natural Compounds Together in the Circle of Trust. Int J Mol Sci 2023;24:6106. https://doi.org/10.3390/ijms24076106.

[43]	Xu F, Ashbrook DG, Gao J, Starlard-Davenport A, Zhao W, Miller DB, et al. Genome-wide transcriptome architecture in a mouse model of Gulf War Illness. Brain Behav Immun 2020;89:209–23. https://doi.org/10.1016/j.bbi.2020.06.018.

[44]	Naviaux RK, Naviaux JC, Li K, Wang L, Monk JM, Bright AT, et al. Metabolic features of Gulf War illness. PLoS One 2019;14:e0219531. https://doi.org/10.1371/journal.pone.0219531.

**Comment 12:**

Data availability: I thought that PLOS ONE’s policy was that all transcriptomic data should be uploaded to GEO?

**Author Response to Reviewer 2 Comment 12:**

*Comment accepted, manuscript modified*.

The authors thank the reviewer for their comment and confirm that the raw data files have been submitted to the National Centre for Biotechnology Information Gene Expression Omnibus database using accession number GSE305935. This will be made publicly available on August 31^st^.

This information is reflected within the data availability statement of the modified manuscript.

**Reviewer #3:**

This is an interesting and valuable study. Very few labs work with human samples from GWI patients. Data analysis was done thoroughly, and conclusions are supported by data.

However, there are some issues.

**Comment 1:**

How did authors deal with genes that had low counts? How background was determined? Was background subtracted?

**Author Response to Reviewer 3 Comment 1:**

*Clarification provided, manuscript not modified.*

The authors thank the reviewer for their comment and hope to provide additional information for clarification. Background correction is completed using negative control probes. The analysis platform, Rosalind Bio, automatically performs negative probe filtering and identifies outliers for removal. Further, subsequent normalisation was not performed using custom settings; however, using default recommended settings. Normalisation was guided by housekeeping probes, which are highlighted within panel details included in Supplementary Material 1.

Details of normalisation are existing within the methods of the manuscript in lines 135-138: “Raw gene expression data was normalised against positive and negative controls. Normalisation and analyses were performed using Rosalind Bio (San Diego, CA, USA) and the following housekeeping genes *ABCF1, ALAS1, EEF1G, G6PD, GAPDH, GUSB, HPRT1, OAZ1, POLR1B, POLR2A, PPIA, RPL19, SDHA, TBP,* and *TUBB* (Supplementary Material 1).”

**Comment 2:**

How healthy controls (HC) were selected? Were HC sedentary? Are/were HCs in military service? As I understand from the manuscript, HCs were not matched with GWI patients by age and BMI. There were no GWI patients younger than 50 years old in 2024. And it is written in the manuscript that participants’ age was from 18 to 65. Definitely, some healthy controls were very young. Do authors consider that age differences between GWI patients and HC could be the reason for some differences in gene expression? The same goes for difference in BMI between GWI patients and HCs. Authors should discuss this issue.

**Author Response to Reviewer 3 Comment 2:**

*Comment accepted, manuscript modified.*

The authors thank the reviewer for the comment, and aligning with Reviewer 2 have included additional information on the impacts of age on the data analysed. Specifically, a general multivariate linear model was used with age as a covariate. The data below demonstrates that the effect of age was non-significant (excluding *IGHV4/59*), thus it is likely that the fold change is reliable across participant ages.

Lines 149-150 of the track changes manuscript: “The effect of age and BMI on gene expression between cohorts was investigated using a general linear multivariate regression model.”

Included in the limitations highlighted within the discussion (lines 429-432) reads: “Given the significant differences in age between the cohorts, a multivariate analysis was performed to determine any potential effect on gene expression. We report that the effect of age was non-significant, excluding one gene being *IGHV4/59* (Supplementary Material 1).”

Furthermore, the impact of BMI was addressed as a limitation within the discussion of the manuscript. A large sample size would enable stratification of BMI according to value, but also category according to the World Health Organization, being underweight, normal, overweight or obese. For transparency in this review, the authors have also determined statistically the potential impact of BMI on the expression of genes across cohort. According to the data below, gene expression may be associated with IGHV4/59 and CEACAM3.

Lines 391-394 of the track changes manuscript: “This is further supported by multivariate analysis undertaken to determine association between BMI and gene expression which found potential associated with the expression of *IGHV4/59* and *CEACAM3* and not genes related to fatty acid metabolism (Supplementary Material 1).”

| Gene | Effect | F | p-value |
| --- | --- | --- | --- |
| TRDV3 | Age | 0.581 | 0.451 |
|  | BMI | 0.829 | 0.720 |
| TRDV1 | Age | 0.406 | 0.529 |
|  | BMI | 7.791 | 0.277 |
| TRDV2 | Age | 0.007 | 0.933 |
|  | BMI | 0.955 | 0.686 |
| TRGC1 | Age | 0.259 | 0.614 |
|  | BMI | 360.14 | 0.042 |
| TRGV4 | Age | 0.251 | 0.620 |
|  | BMI | 0.407 | 0.873 |
| IL7 | Age | 0.281 | 0.600 |
|  | BMI | 140.408 | 0.067 |
| IGHV4/59 | Age | 7.515 | **0.010** |
|  | BMI | 276.352 | **0.048** |
| IGHG1 | Age | 1.488 | 0.231 |
|  | BMI | 70.373 | 0.094 |
| EHHADH | Age | 1.295 | 0.264 |
|  | BMI | 129.61 | 0.069 |
| IDO1 | Age | 1.212 | 0.279 |
|  | BMI | 1.583 | 0.567 |
| CXCR6 | Age | 0.168 | 0.684 |
|  | BMI | 10.872 | 0.236 |
| SESN2 | Age | 0.003 | 0.960 |
|  | BMI | 11.303 | 0.232 |
| SIGLEC1 | Age | 0.061 | 0.806 |
|  | BMI | 3.487 | 0.404 |
| BPI | Age | 0.080 | 0.825 |
|  | BMI | 1.847 | 0.533 |
| MMP9 | Age | 2.657 | 0.113 |
|  | BMI | 1.874 | 0.530 |
| RSAD2 | Age | 0.0 | 0.997 |
|  | BMI | 6.733 | 0.297 |
| CEACAM1 | Age | 1.917 | 0.176 |
|  | BMI | 6.303 | 0.307 |
| CEACAM3 | Age | 0.911 | 0.347 |
|  | BMI | 7843.65 | **0.009** |
| IFIT1 | Age | 0.208 | 0.652 |
|  | BMI | 1.357 | 0.603 |
| IFIT3 | Age | 0.791 | 0.380 |
|  | BMI | 0.713 | 0.755 |
| CXCL1/2/3 | Age | 0.190 | 0.666 |
|  | BMI | 1.426 | 0.591 |
| PTGS2 | Age | 0.917 | 0.345 |
|  | BMI | 181.609 | 0.059 |
| ITGB3 | Age | 0.457 | 0.504 |
|  | BMI | 3.595 | 0.398 |
| EGF | Age | 0.742 | 0.395 |
|  | BMI | 1.134 | 0.645 |
| LTBP1 | Age | 2.048 | 0.162 |
|  | BMI | 12.419 | 0.222 |
| GREM2 | Age | 2.024 | 0.165 |
|  | BMI | 11.737 | 0.228 |
| OAS3 | Age | 0.182 | 0.673 |
|  | BMI | 1.361 | 0.602 |
| IL1B | Age | 0.367 | 0.549 |
|  | BMI | 3.232 | 0.418 |
| ELOVL7 | Age | 0.861 | 0.360 |
|  | BMI | 1.745 | 0.545 |
| MX1 | Age | 0.126 | 0.725 |
|  | BMI | 2.149 | 0.500 |
| MX2 | Age | 0.561 | 0.459 |
|  | BMI | 1.260 | 0.620 |
| TNFAIP3 | Age | 2.391 | 0.132 |
|  | BMI | 0.385 | 0.883 |
| FCAR | Age | 0.499 | 0.485 |
|  | BMI | 0.892 | 0.702 |

**Comment 3:**

Authors should describe more the treatment of participants at the time of blood collection. Were participants fasting? Did they rest before blood collection? This is critical for the comparison of results of different studies.

**Author Response to Reviewer 3 Comment 3:**

*Comment accepted, manuscript modified.*

The authors thank the reviewer for their comment and have included additional information on the time of the blood collection and whether participants were non-fasted. Rest was not a requirement prior to the collection of samples; however, all participants remained seating immediately prior to the collection to obtain informed consent and to process administrative paperwork with the pathology collection centres. Participants were not subjected to physical exertion protocols.

Line 109 of the modified manuscript now includes the following sentence: “All samples were collected between the hours of 7:00AM and 11:00AM from non-fasted participants.”

**Comment 4:**

Authors should add the comparison of their results with the results of transcriptomic GWI studies from other labs, for example, with earlier microarrays results and with the later RNA-seq results on animal GWI model and human GWI transcriptomic data. Direct comparisons (differentially expressed genes) probably are not possible, but comparison on the level of pathways and gene ontologies will strengthen the manuscript.

**Author Response to Reviewer 3 Comment 4:**

*Comment accepted, manuscript modified.*

The authors thank the reviewer for their comment and have since expanded the discussion to incorporate additional references on transcriptomic changes in veterans with GWI or in GWI mice models. To concur with the reviewer, direct comparisons were difficult and there are limited targeted investigations similar to the present investigation to highlight similarities and differences. Instead, literature review found genetic markers across genes linked with environmental exposures and neurodegeneration. These are reflected in the discussion:

Lines 311-323 of the track changes manuscript: “Previous studies have investigated genetic markers in GWI with the identification of nerve agent susceptible genes including *PON1* (paraoxonase-1) and *BChE* (butyrylcholinesterase) [22,23] as well as the neurodegeneration gene *APOE* (apolipoprotein) [24]. Meanwhile, immune profiling has identified altered expression of inflammatory markers including *IL-1β, TNFα, MMP-2, CCL12,* and *EGF*, some of which aligning with the present investigation [25]. A previous study employing logistic regression modelling created a prediction model of GWI risk associated with genetic variability in *TGF* (rs1800469, p=0.009), *IL6R* (rs8192284, p=0.004) and *TLR4* (rs4986791, p=0.013) [17]. While variability within these mentioned genes was not identified in the present study, toll-like receptor cascades and IL signalling were significantly associated with the top differential genes identified in the present manuscript. Further research has also reported an associated with human leukocyte antigen (HLA) allele DRB1*13:02 [26].”

*Included references:*

[17]	Coller JK, Tuke J, Wain TJ, Quinn E, Steele L, Abreu M, et al. Associations of Immune Genetic Variability with Gulf War Illness in 1990–1991 Gulf War Veterans from the Gulf War Illness Consortium (GWIC) Multisite Case-Control Study. Brain Sci 2021;11:1410. https://doi.org/10.3390/brainsci11111410.

[22]	Haley RW, Kramer G, Xiao J, Dever JA, Teiber JF. Evaluation of a Gene-Environment Interaction of PON1 and Low-Level Nerve Agent Exposure with Gulf War Illness: A Prevalence Case-Control Study Drawn from the U.S. Military Health Survey’s National Population Sample. Environ Health Perspect 2022;130:57001. https://doi.org/10.1289/EHP9009.

[23]	Golomb BA, Kelley RI, Han JH, Miller B, Bui L. Gulf war illness: a tale of two genomes. BMC Research Notes 2024;17:230. https://doi.org/10.1186/s13104-024-06871-z.

[24]	Abdullah L, Nkiliza A, Niedospial D, Aldrich G, Bartenfelder G, Keegan A, et al. Genetic association between the APOE ε4 allele, toxicant exposures and Gulf war illness diagnosis. Environ Health 2023;22:51. https://doi.org/10.1186/s12940-023-01002-w.

[25]	Bryant JD, Kodali M, Shuai B, Menissy SS, Graves PJ, Phan TT, et al. Neuroimmune mechanisms of cognitive impairment in a mouse model of Gulf War illness. Brain Behav Immun 2021;97:204–18. https://doi.org/10.1016/j.bbi.2021.07.015.

[26]	James LM, Christova P, Engdahl BE, Lewis SM, Carpenter AF, Georgopoulos AP. Human Leukocyte Antigen (HLA) and Gulf War Illness (GWI): HLA-DRB1*13:02 Spares Subcortical Atrophy in Gulf War Veterans. eBioMedicine 2017;26:126–31. https://doi.org/10.1016/j.ebiom.2017.11.005.
